# Vital Nourishment and Similia

**Published:** 1896-09

**Authors:** William L. Morgan

**Affiliations:** Baltimore, Md.


### VITAL NOURISHMENT AND SIMILIA.

William L. Morgan, M. D., Baltimore, Md.

(Read before the International Hahnemannian Association, June, 1896.)

The earliest history we have of our race is given by Moses, and is supposed to go back to about six thousand years ago. When God created the world and all living things He did not tell how He did it, or what material He used, except that He said He made man out of the dust of the ground. And we would add that at first, after being created, man was still of the ground with an unconscious life as vegetable life, drawing support from the mother earth, incapable of self-support or self-mobility, but with the germ of life, consciousness, and intellectual faculties. When God saw what He intended to be the most perfect of creation lying silent and motionless on the mother ground, He opened the inert nostrils and "breathed into him the breath of life." And then and not till then did "man become a living soul." Then God looked at him and said: "I have created man in my own image."

In the Vedas there are many things corroborative of this statement, excepting the dates, which, in Hindoo chronology, reach many hundred years farther back into pre-historic times, long before the day of stenography or even written languages. Therefore we have reason to give credit to this account. After the knowledge of the manner in which man was created remained in the safe-keeping of the Creator's memory for nearly three thousand years, while men were multiplying, learning, making languages, and inventing writing material, then God appeared unto Moses and made him His amanuensis to record, as He dictated, how it was done so many years before. When we carefully compare the different stages of conception, birth, growth, and maturity of man individually, the woman's physical body, which is composed of the dust of the ground and prepared by the hand of God to receive the conception in the womb—the matrix—which is supplied with the various organic and inorganic elements in proportions, purity, and fineness necessary to be used in building and forming the body, we can see all these elements coming through the living mother's circulation, preparing the embryo when the work is all complete, and at the time fixed by God to take on animal life. It may be observed that in all the period of gestation this new being has no independent life of its own, but is entirely dependent on the life of the mother, as the young fruit is dependent on the parent stalk for its life and growth. The material necessary for this life and growth comes from the dust of the ground through the mother's nourishment and circulation, as God says in Genesis ii, 7: "And the Lord God formed man of the dust of the ground [he was now formed as an image, but not alive and not yet man]. And He breathed into his nostrils the breath of life. [Then] man became a living soul." Thus it appears plainly that man [Adam] had no life or soul till his nostrils were opened and the breath, the air, was forced in and the lungs inflated with atmospheric air, the foramen ovale closed, the circulation established independently of the mother earth, and he became sensible of and sensitive to external contact and impressions, which could not be until all the physical organism had been ready, everything complete, so far as the material is concerned. Then God breathed into his nostrils the breath of life—life from God—and immediately he ceased to be an inert, inactive, ponderous mass of flesh and blood, but a living soul. Thus we may clearly understand what God meant when He said to Himself before man was that He intended to create "man in His own image." He meant man was to be a living soul.

The fœtus in the womb ready to be born is the living organism, but not sensible of or sensitive to external contact or impressions and surroundings. When it passes that critical ordeal of birth, it is not yet a living soul or man. It still lacks the important element for which all these mechanical preparations have been made. And the nostrils are opened for the influx of pure atmospheric air, which gives combustion to the tinder of this delicate organism, and it becomes a "living soul." An automatic, internal spirit, exercising dominion over and giving power to the otherwise inert structure—active spirit, pure and innocent, the image of God.

The reader will hardly fail, from the above, to clearly understand that consciousness, the senses and sensibilities, mental faculties, the power of locomotion and reasoning, commence with the first inhalation of air into the lungs, and continue to perform their functions, except when part of them may be suspended by disease or accident, so long as the breathing continues. But when the nostrils cease to receive and transmit to the lungs the breath of life prepared and kept in readiness in inexhaustible supply—the atmospheric air—then, consciousness is at an end. The senses are gone. The active limbs and brilliant eyes cease to charm, and the process of decay at once commences, and the body returns to the dust from whence it came.

While breathing continues, the breath of life continues to sustain and nourish the living soul—the inner man—that runs the machine, the material man that works the jaws and lips, rules the tongue in masticating the food, opens the ducts, and supplies the digestive fluids, and the entire process of preparing the material necessary to support the external or visible body, the inert part of the being, the tenement for the dwelling of the living soul—the man.

Thus we see according to Revelation that the body, the material part of man, is composed of substance from the ground and the life (soul) is composed of something from the atmospheric air.

It now becomes interesting to view the composition of man according to our own knowledge of things at the present time, and view man as an animal.

There is scarcely a mineral or salt found in the earth which is not found in the animal composition. In the great variety of vegetable and plant foods the same obtains; all substances in the ground are in solution mixed in the soil. The roots of plants ramify in the soil, draw water from it containing the mineral solution which is taken up the stalk to the branches, leaves, flowers, and fruit. By this route it passes through the mysterious processes of refining and adjusting the proper proportions and quantities to make perfect fruit, and after receiving the breath of life through the leaves (the lungs) of the plant, it is then prepared to take on plant life, and to become a part of the stalk or of the fruit, flower, or germ of fruit according to its kind, each according to its own individual species. When matured, it is in readiness to commence the further process of animal digestion. Prepared in Nature's laboratory in exact proportions and qualities, the plant is endowed with that property unknown to material chemistry, but which is plant-life—that property which gives to each species of plant its distinguishing, characteristic differences from each other in growth, taste, and smell, color and uses as animal food or shelter. In this it may be seen that Nature's laboratory is thoroughly equipped, and utilizes re-agents that the modern scientist has yet to discover.

We find in the animal growth a much more complicated but not more perfect chemical arrangement in the process of mastication and digestion, or the process of taking from the plant and fruits the substances suitable to nourish the animal body, which is controlled and managed by the life which is nourished from the air breathed.

The anatomy and physiology is supposed to be well understood, as well as the process of digestion, but the part performed by that occult force called life is as little understood as that of crystallization or the magnet, or how a cathartic causes an action of the bowels, and even less, of how a high potency, low potency, or crude drug can cure.

One ignorant of Homœopathy objects to it, saying: "I cannot believe in it, for I cannot see how such small doses can cure." Ask him if he can tell how large doses cure. He will answer: "I do not know, but I have always been used to that way." Ask him if large doses cure at all. He is apt to answer: "I do not know." Ask the microscopist how a drop of water turns to ice or freezes. He says: "I watch the process, I see great activity in the water, and all at once a crystal formed and then another, and they join together, and so on, but can never see the operation of formation." In that I see the mysteries o Nature working in secret right before my eyes, and yet cannot see them nor comprehend them.

See how mysterious Nature's processes are to the vision of the materialist! But let us compare what is readily seen in the various parts and the effects produced, and take deductions by way of reasoning and find a conclusion as to how it is performed and what agent controls the operation.

If an animal fails to eat food according to its nature, and the nature of the substance from the ground prepared by vegetable digestion and life, or fails to digest it after it is eaten, it will surely dwindle away and die in a longer or shorter time. If it ceases to breathe, life ceases in a few minutes. Thus we see that life cannot exist and govern all the functions of the body without the constant supply of air, and we must understand that the purest air is required, and that impure or adulterated air cannot be nourishing food for life, and that life badly nourished must fail to perform its office. As the air is invisible, the only way that impurity of the life-food can be made known is by the debilitated and deranged condition of the material organism, which is known to indicate a departure from health. Such symptoms are grouped under names of different diseases.

As the air we breathe nourishes life, and after it has been in the lungs and exhaled is then not only unfit to be used again, but really inimical to the natural life, there is therefore a necessity of a continuation of a fresh supply for each succeeding inhalation to insure good health.

Swamps, lowlands, and places shaded from sunlight with vegetable and animal matter in a state of decomposition, produce gases containing many properties that are inimical to life. These gases rise and permeate the air at an altitude regulated to their relative density, and the atmospheric pressure where they perhaps may be and are breathed with, and in place of pure air, producing their debilitating effects called disease upon the system, the miasmatic and malarial diseases of lowlands and marshy places.

This leads us to consider the great variety of lives and life-force, and we see that while these miasms are inimical to human life, they are essential to reptile and many forms of insect and microbe life. The latter cannot live without them. They are like the man in the fable who lived on poisons. They live in and flourish on these emanations that are disease and death to man and many of the higher order of animals. They are their life-nourishment; while they are to man disease-vitality. Hence, we claim among the many vital forces one called (though an apparently contradictory term) disease-vital-force (*Organon*, Section 7), which sustains reptiles and microbes and poisonous plants.

From this it appears plainly that each species of animal has a life-force of its own, which is subject to the encroachment of other forces which carry a blighting and destroying influence (disease) with them. Vegetations of various kinds have such forces, and are nourished by the various miasms that mingle in the air. They grow and flourish, and we call them poisons, and through all history we have used such herbs as medicines. And they are that which we find so valuable in potencies for curing the sick on principles of similia, as the virus, or disease-vital-force of the animal and insect creation. For those morbid, vital principles are by air charged with morbid miasms, which are deadly to man, and other higher animals, but when potentiated become curative in case of sickness.

Do not accuse me of pretending to offer new ideas, for they are not new. We commenced at the creation in sacred history, and we will now come down to a time in history when Jesus the Christ was walking through the tombs and met a lunatic who was a terror to the people, and the devils recognized Jesus and cried out, when He said: "What is thy name?" And they said "Legions, for we are many." By this we see that there were a great number of devils (diseases) in him. The same devils went into the swine and destroyed the whole herd. Perhaps hundreds, and each hog had his devil. Then think how many devils (diseases) may be in one man! And when they were out of him he went his way clothed in his right mind.

Again in his travels, Jesus found some of His disciples trying to cast out a devil and they could not. They appealed to Him, and He said: "This kind can only be cast out by fasting and prayer." In this it may be seen that a variety of devils (diseases) was recognized by Christ. And whenever He cast out devils (healed the sick) they went away well. Even Mary Magdalene, out of whom he cast seven devils, was healed. I suppose it will not be difficult for the reader to see with me the relation between the devils of that day and many nervous and painful diseases of this day.

In the first instance, the lunacy-disease, or devil, was invisible, but intelligent, able to talk and ask permission to go into the swine and make them mad to their own destruction, yet they obeyed the command of our Lord.

Again, in all cases where Christ healed the sick He forgave their sins, and health was restored. This shows that sin, a violation of the laws of health, debilitated the sinner—that sin entered and took up its abode in him, and posted on the outside the sign of the tenant, disease, or devil, with the marks of violated laws of health.

With this view of the "spirit-like vital-force" (natural life), and the spirit-like vital-force of disease, the next thing to be considered is the etiology of disease, and simillimum. Living in places and conditions where the air breathed is contaminated and charged with the vital principles suited to nourish poisonous plants until it is deprived of the natural vital nourishment, and consequently starves the life, and leaves it debilitated, the enfeebled life, after eating the usual amount of material food, is unequal to the work of digesting and assimilating so much material necessary for the building of tissues, and as a consequence great debility follows. In this depraved condition the morbid force designed for other purposes enters and commences its work. If it be that intended for Nightshade, nothing is known of it by smell or taste, but we may know what it is by the symptoms it produces, being a true copy of the Belladonna, and we know it is caused by the same spirit-force that gives the character to that plant. Then in the process of potentiating the juice of the plant, this morbid vitality is set free from its material base and incorporated loosely with the menstruum, and administered to the sufferer. It pervades the system like the spirit that it is. Either by affinity it absorbs the disease-vitality, or by magnetic attraction, or repulsion, or some other undiscovered law of Nature, drives the disease away. This may explain the similitude of any other remedy to disease.

When the disease emanations from other sick subjects mingle with those that have already depressed the vital-force, complications come in ; when the symptoms must be taken into totality and compared with the symptoms of whatever drug will produce such a set of symptoms. By that means we find the drug that contains the morbid force capable of producing morbid symptoms similar to the case to be treated, that sustained and nourished the disease vegetations or microbes in the sick subject —the cause curative action as in the above methods.

Leaving the paludal districts and other unsanitary situations with their proportion of sources of life-giving and nourishing as well as disease or life-destroying properties, let us examine the higher altitudes and celestial regions, and reflect awhile on the situation.

The influence of the great electric system in keeping the atmosphere in a constant agitation, as in winds, whirlwinds, and cyclones, mixing the air of the higher altitudes with that of the lower valleys, driving the foul air from paludal districts and the crowded cities and replacing it with the fresher and purer air from the higher regions, with its rich supply of fresh vital nourishment to be breathed, the vital part consumed, and the residue exhaled and carried away to give place to the ready fresh supply, is evidence of the great perfection of Nature's arrangements. And what the citizens of St. Louis now consider their great calamity is only an extreme action of the great source of health, happiness, and comfort of all living things. As in a vacuum life cannot exist, neither can it in a pit or close room filled with devitalized or once breathed air or foul gases, even disinfectants.

The Rœntgen discoveries, the production of the deeply-penetrating ray in the vacuum (the ether in Crookes' tubes) and the dissolving or separating of the solar rays by the spectroscope, have opened the field for discoveries of the working of the occult forces.

The solar rays, emanating from the great electric centre, pass through the ninety-five million miles of space (ether), come in contact with the outer surface of atmosphere, which is supposed to be so rare that it makes no refraction, but passing further comes in contact with the higher strata of the high white clouds so familiar to our own view, which are composed of frozen mist, each particle of which, being a refracting body, and the cloud of small refracting bodies is dense enough to throw a shadow on the earth. That shadow has an influence on the lower clouds which are not frozen, but are watery mist, and equally capable of dissolving light and separating the rays, as seen in the rainbow. Some of the properties of the different rays (colors) are known by the effects on animals and vegetable growth, and many more are yet to be found out. When clouds are heavy and remain so for many days, the well-known effects on nervous and rheumatic subjects are acutely visible on the mental and physical functions. Plants grow pale and stalks slender. In clear sky, when the solar rays come to the earth unobstructed, all Nature rejoices; mental faculties are clear and all is bright and happy, pains and aches subside, plants take richer, deeper colors, and the stalks grow thicker and stronger. The individual character, as taste, smell, and other properties, are more marked; the animals which, under clouds, were dull and inactive are bright, active, and playful in the sunshine and open field. This should not be considered the direct effect of light, but the changed condition of the air, which will be explained further on.

It has been reported that when the head is exposed to X rays for a considerable time there is produced a very depressed and unpleasant sensation supposed to be injurious. Hence is it not reasonable to conclude that the light coming in contact with the clouds, the brighter rays are refracted and the irrefrangible X, or dark rays, penetrate through the clouds, and deteriorate the life-giving properties, and cause the blighting effects, both seen and felt in animals and plants, rendering both so susceptible to disease (morbid conditions generally). Perhaps the X rays thus formed killed microbes, which are so essential to animal health and comfort, as they do in the culture tubes. And the undivided rays warm the vitalizing properties of the air, expel the surplus moisture, and leave a pure air that nourishes animals, vegetables, and even microbes, and sets free the life-force that sustains health, growth, and especially develops the distinct individuality of species.

During cloudy weather, the density of the air is much reduced, and again the great amount of moisture displaces a large percentage of the air, so that in ordinary breathing the lungs get too much water and not enough of the life-nourishing pure air; while in clear weather there is greater density and less water, and from these conditions, a very greatly increased proportion of pure air warmed and made more volatile or subtle by the sun's light, goes into the lungs and carries with it equally larger amount of dynamus—the life-nourishing principle—which, through the sympathetic system, promotes circulation, digestion, assimilation, and gives both to the muscle and intellect greater activity; in short, health and greater power to resist the encroachments of disease.

Two years ago the irrefrangible X ray was unknown. By an accident it was discovered, yet it had existed from the beginning. Two years ago Argon, an element in the atmosphere, was not thought of, but now it is known. At one time the planets appeared to move strangely to the astronomer, by their movements he calculated the locality of another planet. He searched in that region and found it there. From the signs of the weather, the time of the day, the shapes of the hills, valleys, and track in the mud or snow, the experienced huntsman knows just where to find the game.

From all the symptoms of every kind that indicate a departure from health with the character of each symptom, the skilled homœopathician can find the curative remedy for his patient, and by its use can remove the disease-vital-force that produced such symptoms—that is to say, cure the sick.

From all this we conclude that there is an element in the atmosphere separate from or loosely connected with the material elements—Oxygen, Nitrogen, and Argon. This element is much finer, more subtle, and entirely beyond the ken of any known means of analysis, even more subtle than electricity, magnetism, or gravitation. This force is recognized in Hindoo philosophy. It is perhaps the greatest of all the great forces of Nature, and this element is the food derived from atmospheric air that nourishes plant and animal life as well as the mental and intellectual faculties—the power that moves the physical organism, and when the air is so filled with vapor or impurities of any kind as to displace a large percentage of this force, the starving life becomes weakened, and disease enters and makes itself known by morbid symptoms. The simillimum in high dynamitization (only a vital-force, no material) in small doses properly used, soon removes the intruder, and makes its presence known by the removal of symptoms and the speedy return to normal health (antagonizing vital-forces).

From the above illustrations, the reader may see and embrace the idea how the body is formed, and that the waste substance is replaced by material from the dust of the ground; that Nature has provided ample and proper processes for the preparation and adaptation of that material to such use through plant growth (plant digestion); that chemical or artifical preparations will not answer the purpose; that without life-force the material body is inert; that life cannot be retained in the body and sustained in its office without a constant supply of the vital part of the air; that this vital element being displaced by morbid vital elements in the air breathed renders the system susceptible to disease-action, and disease-action makes itself known by impressions on the organism called signs of dis-ease; that through the vital-force remedies remove disease-vital-force, and aid the natural life in utilizing material matter in restoring the damage done by disease to the organism; that a remedy to be curative must be a spirit-like vital-force similar to the disease-life in the sick; that potentiation of remedies releases the life-force from the material of the drug, and holds it loosely in the menstruum ready for use; that invalids always suffer from a badly nourished vitality and a poorly sustained body.

Hence, for the care and cure of the sick, secure a proper temperature and surroundings; and abundance of pure air to breathe. The true simillimum properly used, and the well-nourished life will utilize the food and attend to nourishing the physical system.

